# SARS-CoV-2 and the Brain: What Do We Know about the Causality of ‘Cognitive COVID?

**DOI:** 10.3390/jcm10153441

**Published:** 2021-08-02

**Authors:** Hashir Ali Awan, Mufaddal Najmuddin Diwan, Alifiya Aamir, Muneeza Ali, Massimo Di Giannantonio, Irfan Ullah, Sheikh Shoib, Domenico De Berardis

**Affiliations:** 1 Department of Internal Medicine, Dow Medical College, Karachi 74200, Pakistan; hashiraliawan@gmail.com (H.A.A.); mufdiwan@gmail.com (M.N.D.); alifiya.aamir521@gmail.com (A.A.); muneeza1998@gmail.com (M.A.); 2 Department of Neurosciences and Imaging, Chair of Psychiatry, University “G. D’Annunzio”, 66100 Chieti, Italy; digiannantonio@unich.it; 3 Department of Internal Medicine, Kabir Medical College, Gandhara University, Peshawar 25000, Pakistan; irfanullahecp2@gmail.com; 4 Department of Internal Medicine, Jawahar Lal Nehru Memorial Hospital, Srinagar 190003, India; Sheikshoib22@gmail.com; 5 NHS, National Health Service, Department of Mental Health, Psychiatric Service for Diagnosis and Treatment, Hospital “G. Mazzini,” ASL 4, 64100 Teramo, Italy

**Keywords:** COVID-19, SARS-CoV-2, brain, neurotropism, cognitive, prevention, diagnosis

## Abstract

The second year of the COVID-19 (coronavirus disease) pandemic has seen the need to identify and assess the long-term consequences of a SARS-CoV-2 infection on an individual’s overall wellbeing, including adequate cognitive functioning. ‘Cognitive COVID’ is an informal term coined to interchangeably refer to acute changes in cognition during COVID-19 and/or cognitive sequelae with various deficits following the infection. These may manifest as altered levels of consciousness, encephalopathy-like symptoms, delirium, and loss of various memory domains. Dysexecutive syndrome is a peculiar manifestation of ‘Cognitive COVID’ as well. In the previous major outbreaks of viruses like SARS-CoV, MERS-CoV and Influenza. There have been attempts to understand the underlying mechanisms describing the causality of similar symptoms following SARS-CoV-2 infection. This review, therefore, is attempting to highlight the current understanding of the various direct and indirect mechanisms, focusing on the role of neurotropism of SARS-CoV-2, the general pro-inflammatory state, and the pandemic-associated psychosocial stressors in the causality of ‘Cognitive COVID.’ Neurotropism is associated with various mechanisms including retrograde neuronal transmission via olfactory pathway, a general hematogenous spread, and the virus using immune cells as vectors. The high amounts of inflammation caused by COVID-19, compounded with potential intubation, are associated with a deleterious effect on the cognition as well. Finally, the pandemic’s unique psychosocial impact has raised alarm due to its possible effect on cognition. Furthermore, with surfacing reports of post-COVID-vaccination cognitive impairments after vaccines containing mRNA encoding for spike glycoprotein of SARS-CoV-2, we hypothesize their causality and ways to mitigate the risk. The potential impact on the quality of life of an individual and the fact that even a minor proportion of COVID-19 cases developing cognitive impairment could be a significant burden on already overwhelmed healthcare systems across the world make it vital to gather further evidence regarding the prevalence, presentation, correlations, and causality of these events and reevaluate our approach to accommodate early identification, management, and rehabilitation of patients exhibiting cognitive symptoms.

## 1. Introduction

Over one year since the first case surfaced in the Chinese city of Wuhan, COVID-19 has resulted in more than 3.7 million deaths globally [1]. Initially, focus was primarily on managing acute conditions, but the long-term consequences of SARS-CoV-2 infection are now being highlighted with time. An interesting example is the patient-coined term ‘Long-COVID’ [2], which denotes long-term outcomes or lasting symptoms of COVID-19 [3].

Apart from major respiratory symptoms, there are reports of acute and post-recovery cognitive deficits occurring in COVID-19 patients [4]. Some authors [5] have also coined a more generic term ‘infectious disease-associated encephalopathy’ to encompass neurological manifestations of both the classical and novel infections. While it is assumed to have a separate pathophysiology than encephalopathy of a non-infectious origin [5], and although evidence of central nervous system (CNS) involvement exists for the 1918 H1N1 Influenza Virus and 2002 SARS-CoV [6], there is a lack of academic evidence necessary to evaluate the causality of cognitive impairments accurately. Nevertheless, several mechanisms have been presented to explain SARS-CoV-2’s acute and ‘sequelae’ effects [7,8,9,10] on the brain. These include viral neurotropism, widespread systemic inflammation, and psychological burden of the pandemic across the world.

These sequelae consist of cognitive impairment after COVID-19 and have also been associated with the medical interventions, especially mechanical ventilation, provided to alleviate conditions of those with severe forms of the infection, which mainly manifested as acute respiratory distress syndrome (ARDS) [11]. Moreover, the immense psychosocial strain due to the prevailing conditions, rising mortality, and government-mandated distancing mechanisms such as lockdowns [12] may also lead to psychological and cognitive consequences in the long run [6].

There has been a critical and time-sensitive [4] need to assess the cognitive impact of COVID-19 due to possible long-term implications it could have on the overall wellbeing of those surviving the infection. This review is attempting to collect the available clinical data, etiological models, and proposed recommendations currently available in the literature to highlight ‘Cognitive COVID’ and determine if it could change our approach in the second year of this global pandemic.

## 2. History of Cognitive Impairment in Previous Major Coronavirus Outbreaks and Other Classical Infectious Diseases

Before SARS-CoV-2, two coronaviruses caused significant outbreaks—the Severe Acute Respiratory Syndrome caused by SARS-CoV in 2002 [13], and the Middle Eastern Respiratory Syndrome caused by MERS-CoV in 2012 [14]. A rapid review published in 2020 highlighted the neurological manifestations of the previous coronaviruses to extrapolate the ratio and predict the number of COVID-19 patients that will potentially show neurological deficits [15].

Furthermore, the comparative pathophysiology of SARS and COVID-19 and a similar psychological strain caused by some of the disease processes and circumstances increase the likelihood that COVID-19 will present with cognitive impairments. SARS and COVID-19 both consist of extensive systemic inflammation, the level of which determines disease severity and outcomes [16]. Furthermore, a study on three MERS patients in Saudi Arabia revealed that they had altered levels of consciousness and confusion, which was correlated to new-onset changes on MRI imaging, indicating a neurological component of the viral infection [17]. Another study on 70 patients in Saudi Arabia found that a quarter of the patients (25.7%) developed confusion during the disease [18].

This link, however, expands beyond just coronaviruses. For example, multiple studies conducted on viral infections involving the Human Immunodeficiency Virus (HIV) and Zika Virus (ZIKV) have also underscored a cognitive aspect to the disease presentation with attention, memory, and learning defects [19,20]. The Influenza viruses have also been reported to affect cognition and result in a cognitive decline. Neurological manifestations of Influenza (NMI) have been reported for both global and seasonal outbreaks of the virus and have ranged from seizures to encephalopathies [21]. A study in Taiwan reported Influenza-associated encephalitis/encephalopathy (IAE) and noted that all 10 patients had different levels of consciousness disturbance on presentation [22]. The prevalence of NMI varies geographically [21] and depends on the dominant viral strain. Rao et al. reported around 18% of all patients of Influenza A (H3N2) in Colorado (USA) had NMI during 2016–17 season [23]. On the other hand, a large national study in Malaysia showed prevalence of NMI to be 8.3% during the 2009 Influenza A (H1N1) pandemic [24]. While the risk of hospitalization is increased by NMI [23], most authors have considered a long-term sequela of such cognitive disturbances by Influenza rare [21,22].

## 3. Brief Review of Manifestation of Acute and Long-Term Cognitive Deficits

Cognitive deficits and impairments have a complex presentation with variable durations [15,25]. In addition, reports for both acute manifestations and long-term sequelae exist [6].

Acute decline in cognitive functions may result due to a combination of causes, including neurotropism of SARS-CoV-2 and sedation during mechanical ventilation. Encephalopathy is then cited as a general cause for the development of cognitive disturbances [26]. Early in the pandemic, a study involving 214 patients in Wuhan, China, noted CNS-related symptoms including dizziness, headache, and diminished consciousness in 24.8% of patients [27]. In April 2020, ‘altered mental status’ was listed as one of the ‘clinical syndromes’ associated with COVID-19 and defined as an ‘acute alteration in personality, behavior, cognition, or consciousness’ by a survey in the United Kingdom [28]. In the same survey, 31% of the patients recorded having an altered mental status following COVID-19, and nearly 5% of the total patients had dementia-like cognitive symptoms [28]. In addition, viral encephalitis has been identified in some COVID-19 patients, and it alone is possibly linked to the development of acute and lasting cognitive losses [6,29].

‘Dysexecutive syndrome’ is another peculiar concept that depicts cognitive defects in individuals, particularly of attention, control, and orientation loss [30]. Empirical evidence from a French study shows that loss of executive functions was reported in almost a quarter of COVID-19 patients presenting with ARDS [31]. Furthermore, there is promising evidence of even asymptomatic COVID-19 subjects scoring significantly lower in domains of visual perception, naming, and fluency when checked via the Montreal Cognitive Assessment (MoCA) test [32]. A review also noted symptoms more prevalent in older individuals and those with severe infections [6].

Apart from lasting psychiatric conditions, cognitive impairments may follow a SARS-CoV-2 infection, causing impaired memory, confusion, and attention deficits in the long term [6,33]. A study in Zhejiang, China administered multiple tests evaluating attention, memory, executive function, and information processing, checking for cognitive function of recovered COVID-19 patients against a control group, and finding the sustained attention domain significantly lesser in COVID-19 survivors [34]. Further exploring the link between hospitalization mostly with mechanical ventilation and cognitive deficits, a study using the BMET was conducted on 57 recovering patients with severe disease [4]. In total, 81% of the cohort exhibited some form of cognitive impairment; however, there was no significant correlation of such deficits with the length of intubation.

Further evidence of long-term deficits is available in two more studies [35,36]. First, Lu et al. [35] recorded data of 60 patients during acute SARS-CoV-2 infection and at a 3-month follow-up visit. The proportion of patients with memory loss more than doubled from 13.3% during the acute disease to 28.3% at the follow-up [35], demonstrating the long-term impact of COVID-19 on an individual’s cognition. In addition, Woo et al. [36] investigated the cognitive status of 18 recovered patients using the Modified Telephone Interview for Cognitive Status (TICS-M). They contacted the patients at a median of 85 days following their recovery from mild or moderate COVID-19 without an ICU admission. Results showed 18 post-COVID-19 patients scoring appreciably lower than ten control patients on the cognitive assessment, with multiple other self-reported cognitive impairments, including attention deficits (50%), memory deficits (44.4%), and incoherent thoughts (5.6%) [36].

## 4. Causality

Owing to the nascency of the novel coronavirus causing the pandemic, the exact pathophysiology behind the cognitive sequelae has not been entirely understood. There is no clarity regarding SARS-CoV-2 *directly* affecting the brain or the symptoms resulting from the non-specific and indirect causes; for instance, systemic inflammation and medical interventions such as ventilation. Additionally, the piling psychosocial strain could also potentially act as the source of ‘Cognitive COVID’ [6,9].

**Figure 1:**
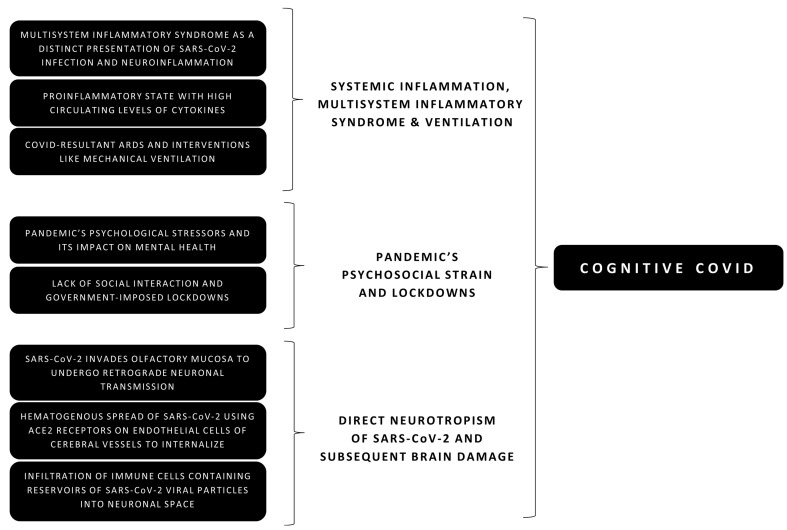
Summary of possible causal elements in the development of cognitive symptoms during and after a COVID-19 (coronavirus disease 2019) infection.

The evidence so far is inconclusive of whether each aspect works solitarily or all elements are working together in causing symptoms. This section briefly outlines these mechanisms (also shown in Figure 1) with references to available evidence of SARS-CoV-2 and its predecessor coronaviruses.

### 4.1. Neurotropism and the ACE2 Receptor

While still unclear, it is hypothesized that SARS-CoV-2, similarly to other coronaviruses, can infect and survive in nervous tissue [37,38]. Although rare, evidence of SARS-CoV-2’s presence in cerebrospinal fluid (CSF) [29,39], as with other viruses [40], is available. There are numerous suggested pathways by which such neurotropism occurs. However, the exact mechanism is still uncertain. Retrograde neuronal access via peripheral nerves, hematogenous spread via directly infecting endothelial cells, and infiltration of infected cells are three main explanations [7,41,42,43] behind how respiratory viruses (such as SARS-CoV-2) enter the CNS.

- Olfactory invasion: There is emerging evidence of SARS-CoV-2 affecting the olfactory and gustatory sensations, producing well-known symptoms of ‘loss of taste and smell’ in infected individuals [44,45,46]. With time, evidence has surfaced supporting the pathobiology of olfactory and gustatory dysfunction because of a direct invasion of the mucosal epithelium and olfactory bulb [47]. The invasion can potentially be attributed to their expression of the ACE2 surface receptor and Transmembrane Protease Serine 2 (TMPRSS2), cleaving the spike protein of SARS-CoV-2 and facilitating the fusion of SARS-CoV-2 with cellular membranes [48,49]. Furthermore, having a genome that is 79% similar to that of SARS-CoV, the spike glycoprotein of SARS-CoV-2 also binds to Angiotensin-Converting Enzyme 2 (ACE2) receptor on multiple organs, including the brain, acting as the viral functional receptor [50,51]. However, SARS-CoV-2 binds to ACE2 receptors with a considerably greater affinity than SARS-CoV [52]. Animal studies focusing on SARS-CoV have shown trans-neuronal spread from the olfactory bulb to certain ‘connected’ regions of the brain, providing key ‘circumstantial evidence’ in the potential neurotropic properties of SARS-CoV-2, as well [43,53]. The entorhinal cortex and the hippocampus are such ‘connected’ regions. They are involved in episodic memory and other domains, illustrating how damage directed at these areas may cause lasting cognitive dysfunction [54].
- Hematogenous spread: Some authors [8] claim hematogenous spread via the cerebral vasculature plays a more critical role in direct brain entry and damage-causing cognitive deficits in COVID-19. Evidence of SARS-CoV-2’s presence in blood samples of some confirmed COVID-19 patients exists. As many as 41% [55] of the samples showed viremia [43], showcasing the ability of the virus to easily reach the brain once the blood-brain barrier (BBB) is damaged. The distribution of SARS-CoV-2’s functional (ACE2) receptor is widespread in endothelial cells and pericytes throughout the body [56]. Analysis of available genomic databases confirms noteworthy expression of the receptor in neuronal and glial tissues of the CNS [56]. Consequently, the nervous tissue is potentially vulnerable if the virus comes in direct contact and interacts with the ACE2 receptors. In addition, SARS-CoV-2’s potential neurotropic properties may allow it to assume latency inside neuronal tissue of patients even after recovery from COVID-19, putting them at greater risk of long-term or delayed cognitive deficits and neurological symptoms [6]. Notably, it is still unclear how abundantly ACE2 receptors are expressed in the cerebral vasculature. However, other docking receptors, importantly basigin (BSG) and neuropilin (NRP1), have been identified as facilitators of the viral entry or internalization—making the brain vulnerable to viral inflammation even with an intact BBB [7] In addition, SARS-CoV-2 and the accompanying inflammatory cytokines, including Interleukins (IL) and Tumor Necrosis Factor (TNF), may damage the BBB [57]. Moreover, evidence shows that SARS-CoV-2 affects vasculature integrity by direct viral infection, leading to endothelium damage and increased vascular permeability in peripheral vessels [58]; extrapolated from the cerebral endothelial cells, this could explain the disruption of the BBB. Therefore, immune-mediated action or direct inflammation may be responsible for endothelial dysfunction in the BBB, enabled by the recruitment of host immune cells. Additional factors that may aid in the hematogenous spread of SARS-CoV-2 to the brain include a pre-existing or underlying neurological pathology and entry via circumventricular organs such as the median eminence of the hypothalamus [7].
- Infiltration of infected cells: A 2005 study aimed at SARS-CoV found a sizeable proportion of immune cells (29.7% of monocytes and 51.5% of lymphocytes) in 6 out of 22 patients to contain viral particles [59], signaling their potential as a reservoir for the virus. If immune cells were to infiltrate the neuronal space by crossing the BBB, this would allow the viral particles in them to cause direct brain damage by binding to ACE2 receptors on neuronal and glial cells [7]. However, whether these findings can be accurately extrapolated to SARS-CoV-2 remains yet to be ascertained. In addition, autopsies and studies conducted on samples obtained from infected individuals have been inconclusive about direct immune cell infiltration during COVID-19 [60].

### 4.2. Non-Specific Systemic Inflammation, Multisystem Inflammatory Syndrome (MIS), and ARDS

- Widespread systemic inflammation: A significant increase in inflammatory cytokines plays a role in SARS symptoms, with inflammation persisting even after the viral clearance, and a similar ramped up an innate immune response in the form of ‘cytokine storm’ is behind COVID-19 as well [16,34,61]. Highly circulating amounts of Interleukins and other mediators (including IL-6, IL-1β, and TNF, and others) resulting in a pro-inflammatory status are commonly found in COVID-19 patients [62,63]. This amplified immune response may cause increased vascular permeability and vasculopathy arising from disseminated intravascular coagulation (DIC). Subsequently, the BBB is compromised, allowing cytokines to activate a microglial inflammatory response [64]. This mechanism may potentially lead to delirium and seizures due to an immune-mediated encephalopathy [6]. There is a substantial risk of Cerebral Vascular Disease in infected individuals potentially due to this exact pathophysiology, with studies showing increased incidences of hypoxic-ischemic conditions [8]. A study in April 2020 investigated the histopathological changes during autopsy, and all 18 patients’ brain specimens depicted hypoxic changes [60]. It also drew attention to how cerebral white matter is at high risk for damage due to ischemia, manifesting as loss of vital cognitive functions during and after COVID-19 [8]. Several studies investigating Alzheimer’s Disease (AD) patients found a notable inflammation in patients showing cognitive deficits compared to the control group, indicating the link between the development of cognitive impairment and increased inflammatory molecules [65]. In addition, previous studies have highlighted the long-term detrimental effects of severe inflammation on the cognitive ability of a person, especially those already with or at high risk of developing a neurodegenerative disease [66,67,68]. A study investigated links between serum inflammatory markers and C-reactive protein (CRP) in COVID-19 patients with cognitive functions and found loss of some domains, such as sustained attention, to be significantly correlated to CRP levels in the blood [34]. In addition, previous longitudinal studies have confirmed a significant association between CRP levels and cognitive decline [69], affirming how underlying inflammation (using CRP as a marker) likely affects an individual’s cognitive functioning in the long run. Some studies have claimed the role of NLRP3 inflammasome activity in exacerbating systemic inflammation and its outcomes [16]. In addition, some proteins of SARS-CoV have shown to induce NLRP4 inflammasome activity, making it likely that SARS-CoV-2 also utilizes similar pathways to cause extensive inflammation [70]. This pathway has further been suggested to explain cognitive deficits due to high IL-1β activity in the setting of hypercapnia caused by mechanical ventilation [71].
- Multisystem Inflammatory Syndrome (MIS): Demographically, COVID-19 has been shown to cause more severe disease in adults, but increasing reports of COVID-associated Multisystem Inflammatory Syndrome (MIS) have surfaced [72,73,74]. While more prevalent in children, as MIS in children (MIS-C), it can potentially occur in adults as well (MIS-A). A meta-analysis comparing MIS-C’s clinical course to COVID-19 revealed how it can potentially lead to multi-organ failure [75]. MIS-C was also shown to have a relatively higher incidence of neurological manifestations compared to acute COVID-19 [75]. As a distinct manifestation of a SARS-CoV-2 infection even in adults [76], with a high risk of neurological symptoms, MIS warrants discussion as a potential causal factor in the development of Cognitive COVID. MIS-C is considered to cause a hyperinflammatory shock and resembles Kawasaki Disease (KD) [77] or Toxic Shock Syndrome (TSS) [78]. Several cases with serologic evidence of a SARS-CoV-2 infection reported symptoms of MIS-C such as shock, cardiac symptoms, gastrointestinal complains, and elevated markers of inflammation, particularly after it was recognized by the Centers for Disease Control and Prevention (CDC) in May 2020 [78]. The pathophysiology of MIS-C during and after a SARS-CoV-2 infection is largely unknown [78]. Generally, MIS-C is believed to cause a dysregulated immune response possibly by viral mimicry of the host and development of autoantibodies. This leads to widespread systemic inflammation that potentially has a damaging impact on multiple systems, including the neurological system [79,80,81]. Interestingly, some cases depicted a milder, ‘overlapping’ syndrome with acute COVID-19, while other cases reported MIS-C symptoms weeks after an acute infection. However, children with an active COVID-19 infection confirmed via a positive RT-PCR test form only one-third of the total MIS-C cases, with a majority showing evidence of a past infection confirmed via serological tests [80]. Jiang et al. uses epidemiological data from different countries to suggest that MIS-C is more likely caused by an acquired, albeit dysfunctional, immune response to SARS-CoV-2 instead of direct viral involvement [80]. The above discussion on widespread systemic inflammation in severe COVID-19 in adults is of value here when discussing MIS-C or MIS-A. The pathophysiology of MIS-C is also believed to involve a cytokine storm with elevated inflammatory mediators [80,82] which may ultimately lead to neurocognitive manifestations, as elucidated previously in this text.
- Acute respiratory distress syndrome (ARDS), mechanical ventilation, and associated cognitive decline: Although the exact ratio of COVID-19 patients developing severe disease and requiring hospitalization or intensive care unit (ICU) admission varies extensively, there is undoubtedly a noticeable proportion that progresses to life-threatening conditions [83]. Preliminary studies from China investigating data of more than 70 thousand patients suggested that around 19% of patients with COVID-19 develop severe or critical disease, most likely necessitating hospitalization [84]. A survey of 17 studies examining statistics of hospitalized COVID-19 patients from different regions found that one-third of all hospitalized and three-quarters of all ICU-admitted patients develop ARDS [83]. Cognitive impairment following ARDS of variable etiology is widely reported and reviewed [85]. Although severe inflammation, hemodynamic instability, and hypoxia have been indicted, the exact mechanism causing it is unknown. However, a review of studies has shown that cognitive impairment post-ARDS has a high incidence and ranges from 70–100% at hospital discharge, to 46–80% at one year after discharge, to 20% at five years after discharge [85]. In addition, an observational study in France described several ICU-admitted COVID-19 patients with complaints of ARDS developing encephalopathy manifesting as confusion and agitation [31]. According to Tzotzos et al., of the COVID-19 ICU-admitted patients who develop ARDS, more than 80% must receive mechanical ventilation [83]. Mechanical ventilation, regardless of ARDS, is associated with cognitive decline and reduced quality of life in the long run [86]. Since mechanical ventilation inextricably leads to the administration of sedatives, it is essential to note delirium and other cognitive consequences that may accompany, both in the short and long term [87]. The likelihood of a systemic inflammation playing a significant role in the development of cognitive loss compared to direct viral damage is underscored by the sparse evidence of the virus being found in the CSF [16]. Furthermore, instead of being two entirely independent processes, the neurotropism of SARS-CoV-2 and the widespread parallel inflammation may also operate in conjunction [7] and collectively lead to direct and indirect neuronal damage with cognitive deficits. Lastly, it is crucial to not trivialize non-specific but potentially key elements in developing cognitive sequelae, namely COVID-19 complications such as ARDS and subsequent mechanical ventilation [11].

### 4.3. The Psychosocial Strain of the Pandemic and Associated Lockdowns

- Psychological stressors: While countries battle their second or third waves, confinement due to lockdowns and the fear of one or one’s loved ones contracting COVID-19 are just some of the reasons that continue to cause an unprecedented psychological burden on people across the world [12,88]. With psychological conditions such as anxiety and depression now being recorded globally, cognitive consequences can be reasonably expected as a unique symptomatic presentation [89]. A systematic review remarked that some studies had shown the prevalence of post-traumatic stress disorder (PTSD) ranging from 7% to as high as 53.8% during the pandemic [90]. Moreover, this psychological disorder has been correlated with diminishing cognitive function, especially in the elderly [91], showing how ‘Cognitive COVID’ is possibly related to an individual’s psychological state.
- Social isolation and government-mandated lockdowns: An article published in late 2020 had reviewed the available evidence and stipulated that social distancing/isolation and lack of human interaction may have a detrimental effect on a person’s cognition [92]. Echoing these findings, a study conducted in Italy during May 2020 investigated the effects of psychological stressors as a result of isolation in the form of national lockdown as a mitigation technique on the global cognitive function of the public [93]. Findings suggested cognitive function such as barring memory deteriorated during lockdowns. Furthermore, with a greater prevalence of anxiety, depression, and other mental health changes, a significant deleterious impact on cognitive function(s) was noted in those who had lesser social interactions [93].

Further research needs to correlate lockdowns and various psychosocial factors of the pandemic with cognitive ability to gather experimental evidence. Consequently, the findings may aid in ascertaining if this psychological burden is responsible for the reason why COVID-19 survivors may develop cognitive sequelae following their recovery. Likewise, any significant conclusions may also illustrate if psychosocial distress in the wake of lockdowns increases an individual’s risk of being affected and the severity by which they are affected due to other causal factors outlined in this text earlier.

## 5. COVID-19 Vaccination, Autoimmunity, and Cognitive Impairment

The vaccine roll-out for COVID-19 began recently, but nearly 3.57 billion doses have been administered worldwide already [1]. Various vaccines were approved by the World Health Organization (WHO) for emergency use but all of them fall under three major subtypes: messenger RNA (mRNA), viral vector, and inactivated whole-virus [94]. As the pace of vaccine administration increases, more data is surfacing regarding post-vaccination adverse events. Neurocognitive symptoms following vaccinations COVID-19 vaccinations are rare but emerging case reports require due attention to accurately evaluate the pathophysiology and risk-factors carefully and accurately.

Two cases of encephalopathy within one week following inoculation via an mRNA vaccine were reported in patients with no prior neuropsychiatric history [95]. Furthermore, an 89-year-old patient developed delirium after a first dose of an mRNA vaccine [96]. The mRNA in the vaccines encodes antigen S-2B, which includes SARS-CoV-2 spike glycoprotein. The spike glycoprotein during a viral infection initiates a cascade of inflammatory reactions after attaching to ACE2 receptor, leading to COVID-19 encephalopathy [95]. Authors hypothesize that cells translating this vaccine mRNA may produce the same glycoprotein and in turn mimic the encephalopathy caused by an active viral infection [95]. Furthermore, acquired immunity via anti-spike antibodies linking to spike protein of SARS-CoV has been known to boost inflammation by activating macrophages [80]. A similar mechanism following development of anti-spike antibodies against SARS-CoV-2 after administration of mRNA vaccines may lead to widespread inflammation, macrophage activation, and development of neurological symptoms [80,95,96].

An interesting case is of an adult, who recovered from COVID-19 6 weeks ago, developing MIS following a second dose of an inactivated virus vaccine [97]. Features of shock and cardiac dysfunction were present in the patient along with elevated inflammatory markers, indicating MIS. The authors postulate that the vaccine may have accentuated their body’s immune response which was ‘already primed’ following SARS-CoV-2 infection and therefore led to an uninhibited inflammatory condition in the body [97].

Some authors have warned against the use of certain immunogenic proteins of SARS-CoV-2 in vaccines that are homologous to the human immune system [98]. With most of SARS-CoV-2’s immunogenic epitopes matching human proteins, there is a reasonable risk that vaccines containing these epitopes will lead to autoimmunity [98]. Excessive inflammation, creation of autoantibodies, and a series of biochemical processes due to autoimmunity may lead to neuroinflammation, damage to neuronal integrity and cognitive impairment [99]. Using Alzheimer’s Disease as a parallel in mouse models, a temporal association between increasing autoimmunity and declining cognitive competence was found [100], highlighting the damaging effect accelerated autoimmunity may have on brain function.

## 6. Discussion

We have identified three major areas of discussion when debating on causality of cognitive symptoms occurring during and after a SARS-CoV-2 infection. The direct neurotropism of SARS-CoV-2 is largely based on information available for SARS-CoV. Several hypotheses on mechanisms of direct neurotropism have been outlined, such as infiltration of virus-laden immune cells, hematogenous spread through CVOs or breaks in BBB, and retrograde neuronal transmission through invasion of the olfactory system. Furthermore, non-specific systemic inflammation which also manifest as multisystemic inflammatory syndrome (MIS) due to a ‘cytokine storm’ reported in COVID patients predisposes them to vascular injuries, leading to neuroinflammation. As mentioned earlier, treatment methods such as intubation to treat ARDS can also lead to cognitive symptoms. Lastly, a neuropsychiatric vantage point allows us to underscore the importance of prevailing psychological stressors and their effect on a person’s cognitive ability.

For the first time, to our knowledge, in this text we have also reviewed case studies of cognitive impairment and adverse-events following COVID-19 vaccinations, especially the mRNA subtype. We have discussed the occurrence of a heightened immune response to spike glycoprotein encoded by the mRNA in the vaccine which leads to a condition similar to COVID-19 encephalopathy. By discussing the potential occurrence of autoimmunity following inoculation, we have identified the risk of using viral epitopes that are homologous to proteins in the human immune system.

Furthermore, despite the varying prevalence and presentations of cognitive deficits, due to the sheer scale of the pandemic, with global cases crossing 173 million [1], any proportion will result in substantial implications on health systems and a massive influx of patients with cognitive complaints. Therefore, with more significant evidence through research, greater awareness regarding the probable emergence of ‘Cognitive COVID’ in some patients is required—both for the public, early seeking medical care, and healthcare workers, for readiness and early detection.

As described above, the varying levels of cognitive impairment will require a thorough evaluation, planned follow-ups, and in-patient management if required. Consequently, facilities and institutions should allocate adequate resources and enable their healthcare workers via training to effectively respond to those COVID-19 survivors at high risk for developing cognitive sequelae [6].

Additionally, the therapeutic significance of understanding and ascertaining the etiology of ‘Cognitive COVID’, particularly on a cellular level, is manifold. There are available interventions that may mitigate the negative impact of high inflammation levels with a potential cytokine storm, including cytokine antagonists and other anti-inflammatory modulators [64]. Importantly, due to the requirement of physical distancing to avoid transmission of SARS-CoV-2, telemedicine for diagnosis and cognitive rehabilitation is an exciting and promising avenue to be utilized [101]. Moreover, as a psychosocial burden and mental health disorders are possible causal elements, telepsychiatry services can play a crucial role in preventing cognitive impairments [102].

## 7. Conclusions, Limitations and Way Forward

Cognitive COVID is an oft-ignored aspect of the pandemic, but with greater attention now being paid to the non-respiratory and long-term cognitive consequences of COVID, it is vital to collect further evidence regarding the prevalence, presentation, correlations, and causality of these events. Furthermore, the potentially long-term nature of these deficits and their devastating effect on quality of life, especially the elderly, makes it more pressing to review our current approach in early identification, management, and rehabilitation of patients exhibiting cognitive symptoms [6]. In this article, we have highlighted the probable causality of Cognitive COVID by reviewing the available hypotheses, case reports, and clinical data that has been published after the pandemic started and used previous coronaviruses as a basis to form a parallel to the novel coronavirus 2019.

Due to the relative recency of the pandemic and unavailability of coherent data, it is extremely challenging to reach a plausible conclusion regarding the intricate interplay of causal factors. Criteria to determine causality such as the Bradford-Hill criteria [103] are difficult to apply on the causal factors. The lack of consistent data available from different regions across the world and various methods of measuring inflammation or cognitive deficits disallow direct comparison. The biological plausibility criterion of Bradford-Hill criteria, however, has been discussed at depth in the text to decipher neurological and inflammatory mechanisms that lead to clinical symptoms. Another criterion of analogy can be extrapolated to use of available data from SARS-CoV and MERS-CoV to predict SARS-CoV-2’s effect on the brain. We are also limited by a lack of research conducted into different variants of SARS-CoV-2 and their effect on cognition.

The way forward is to develop a standardized protocol for neurocognitive assessment of COVID-19 patients, especially at times of discharge from hospitalization and end of medical interventions such as intubation. These steps will mitigate the threat posed by ‘Cognitive COVID’ and will undoubtedly decrease the burden on already overwhelmed healthcare systems.

Moving forward, greater attention should be paid to cognitive impairment during and after COVID-19 and vaccination. With the emergence of new strains of COVID-19, such as the Delta and the Lambda variant [104], the variation in prevalence of cognitive manifestations of the viral infection needs to be ascertained. Therefore, it is imperative to collect empirical data from multiple demographics in order to attain uniform clinical and biochemical information regarding causality and risk-factors in developing cognitive impairments.

## Data Availability

No new data were created or analyzed in this study. Data sharing does not apply to this article.
